# Blood pressure trends and disparities across the COVID-19 pandemic in a large diverse urban population

**DOI:** 10.1038/s41371-026-01130-z

**Published:** 2026-03-13

**Authors:** Vincent Zhang, Stephen H. Wang, Kevin Fiori, Wei Hou, Katie S. Duong, Sonya S. Henry, Lili Zhang, Tim Q. Duong

**Affiliations:** 1 Department of Radiology, Albert Einstein College of Medicine and Montefiore Medical Center, Bronx, NY USA; 2 Icahn School of Medicine at Mount Sinai, New York, NY USA; 3 Department of Surgery, Beth Israel Deaconess Medical Center and Harvard Medical School, Boston, MA USA; 4 Department of Pediatrics, Albert Einstein College of Medicine and Montefiore Medical Center, Bronx, NY USA; 5 Department of Medicine, Division of Cardiology, Albert Einstein College of Medicine and Montefiore Medical Center, Bronx, NY USA; 6 Center for Health & Data Innovation, Albert Einstein College of Medicine and Montefiore Medical Center, Bronx, NY USA

**Keywords:** Risk factors, Preventive medicine

## Abstract

The COVID-19 pandemic disrupted healthcare systems, daily routines, and socioeconomic conditions, potentially increasing systolic blood pressure (SBP) at the population level. Understanding long-term SBP trends is critical to inform hypertension management and address pandemic-exacerbated disparities. We performed a retrospective study using interrupted time series analyses of electronic medical records from the Montefiore Health System (January 2017–August 2024). Adults with in-person outpatient visits were included in the primary analysis of comparing SBP trends post-onset of pandemic to pre-pandemic. Secondary analyses stratified by age, sex, race/ethnicity, and neighborhood-level socioeconomic indicators. Among 789,897 patients with 8,207,177 outpatient SBP measurements (55.0 ± 18.4 years old; 66.5% female), SBP increased by 1.69 mmHg (95% CI, 1.59-1.78; P < 0.001), and did not return to pre-pandemic levels until 16 months later. Racial/ethnic minorities experienced larger pandemic-related SBP increases (P < 0.05). Patients from lower socioeconomic neighborhoods had greater SBP increases than residents of higher socioeconomic neighborhoods (P < 0.05). The COVID-19 pandemic was associated with sustained population-level increases in SBP, disproportionately affecting racial/ethnic minorities and lower socioeconomic groups. These findings highlight the need for targeted interventions to mitigate long-term cardiovascular risks and reduce exacerbated health disparities.

## Introduction

The COVID-19 pandemic represented an unprecedented disruption to healthcare systems worldwide and posed particular challenges for the management of blood pressure. Early in the pandemic, multiple studies reported short-term increases in population-level blood pressure and worsening hypertension control, raising concerns about the pandemic’s potential long-term cardiometabolic consequences. However, the durability of these changes and whether blood pressure levels eventually returned to pre-pandemic trends remain incompletely understood.

The pandemic substantially altered healthcare delivery and disrupted routine outpatient care for millions of Americans. For patients with hypertension—who rely on regular blood pressure monitoring, medication titration, and continuity of care—these disruptions may have affected disease management [1, 2]. Even more, multiple studies suggest delayed diagnosis of new hypertension cases due to the disruption of outpatient primary care visits [3].

Additionally, the pandemic exacerbated financial, social, emotional, lifestyle, and other stressors that may have led to transient rises in blood pressure [2, 4]. Emerging evidence also suggests that pandemic-related stressors and disruptions in care were not experienced uniformly, with greater impacts among racial and ethnic minority populations and individuals with lower socioeconomic status—as reflected by income, education, employment, and neighborhood context [5, 6].

Long-standing disparities in hypertension prevalence, control, and outcomes among racial and ethnic minorities and socioeconomically disadvantaged populations are documented well before the beginning of the pandemic [7, 8]. In the United States, non-Hispanic Black and Hispanic populations experience higher rates of hypertension and lower rates of blood pressure control compared with non-Hispanic White populations. Similarly, lower socioeconomic status is associated with a higher burden of hypertension, poorer access to care, and worse cardiovascular outcomes [9, 10].

A few studies have documented short-term, population-wide increases in blood pressure in the United States, the United Kingdom, and Australia [11–14], raising concerns about the pandemic’s potential to cause lasting changes in blood pressure trends. However, evidence on the pandemic’s long-term impact remains limited. It remains unclear whether increases in systolic blood pressure (SBP) were sustained or transient, and if transient, when SBP might return to pre-pandemic levels. Moreover, it is unclear the impact of the pandemic on racial/ethnic and socioeconomic disparities in regard to blood pressure trends.

The goal of this study was to examine in-person outpatient SBP trends at the population level across the COVID-19 pandemic in an diverse, urban population from January 2017 to January 2024. We employed interrupted time series (ITS) analysis to compare pre- and post-onset of the pandemic periods, allowing assessment of deviations from underlying secular blood pressure trends. Specifically, we sought to determine how much SBP deviated from pre-pandemic trends following pandemic onset, whether and when these trends returned to levels predicted by pre-pandemic trajectories, and whether pandemic-associated changes differed across demographic groups and neighborhood-level socioeconomic strata. Our data came from the Montefiore Health System which serves a large diverse population in the Bronx, an epicenter of early COVID-19 pandemic and subsequent surges of infection. This cohort was one of the first groups to experience SARS-CoV-2 infections [15] and these analyses offer a first glimpse of potential long-term risks for elevated blood pressure at the population level and potential disparities in these trends.

## Methods

### Study population

Health data came from the Montefiore Health System - one of the largest healthcare systems in NYC, with 10 hospitals and 200 outpatient ambulatory clinics primarily located in the Bronx, Westchester, and the Hudson Valley, and serving approximately 3 million patients annuals. The system serves a large, low-income, and racially and ethnically diverse population that was heavily impacted by COVID-19 early in the pandemic and subsequent waves of infection.

Montefiore Health System’s Epic electronic health record data were extracted automatically between January 2017-August 2024 as described previously [16–18] and patients were identified by deidentified person-id based on Medical Record Number (MRN) provided by data brokers. Adults (≥18 years) with at least one in-person outpatient systolic blood pressure (SBP) measurement recorded in the Montefiore Health System between January 2017 and August 2024 were eligible for inclusion (see Supplemental Fig. 1 for study flowchart). Only in-person outpatient visits were included in our study to avoid potential confounding of inpatient SBP from various severe illnesses. Visits from all specialties were included. Patients entered the analytic cohort at the time of their first qualifying outpatient SBP measurement during the study period and could contribute repeated observations over time. A total of 789,897 number of unique patients contributed 8,207,177 unique outpatient SBP measurements during this time period.

### Outcome variables

The primary outcome was systolic blood pressure (SBP), obtained from in-person outpatient clinical measurements recorded in the EHR. SBP measurements were first aggregated at the patient-month level by calculating the mean SBP for each patient within each calendar month, such that each patient contributed at most one SBP value per month.

To characterize the distribution of SBP values and minimize the influence of extreme measurements, percentile thresholds at the 0.1th and 99.9th percentiles were used as cutoffs (Supplemental Fig. 2).

### Independent variables and covariates

The primary independent variable was pandemic onset, modeled as a binary indicator representing pre- versus post-onset of pandemic (March 2020) periods.

Demographic covariates included age at time of SBP measurement, sex, and self-reported race and ethnicity. Pre-existing comorbidities included asthma, chronic obstructive pulmonary disease (COPD), diabetes mellitus, chronic heart failure (CHF), hypertension (HTN), chronic kidney disease (CKD), cardiovascular diseases (CAD; coronary artery disease, ischemic heart disease, and angina), obesity, and hyperlipidemia (HLD) as defined by ICD-10 diagnostic codes. Status of comorbidities was determined at each SBP measurement time point.

The 2021 5-year average of median income data, education (high school graduation aged 25 and above), and unemployment rates were obtained by matching the patient’s Zone Improvement Plan (ZIP) code to its median household income/unemployment/education rate as reported by the United States Census Bureau American Community Survey (https://data.census.gov). Median income, education, and unemployment rates per zip code were grouped into quintiles based on patient population with SBP data. For sake of consistency, higher quintiles are associated with higher SES status (e.g. higher median income, higher education rate, and lower unemployment rate). Neighborhood-level socioeconomic data were available for approximately 98–99% of observations, with missingness primarily due to incomplete or invalid ZIP code information in the electronic health record. Missing socioeconomic data were not used as exclusion criteria for cohort construction. Instead, analyses incorporating these variables were conducted using complete-case observations for each model, resulting in varying sample sizes across sub-analyses. The number of observations included in each analysis is reported in the corresponding tables and figures.

### Statistical methods and data analysis

SBP measurements were first aggregated at the patient-month level by calculating the mean SBP for each patient within each calendar month. These patient-month observations were then used as the unit of analysis in the interrupted time series models, such that patients could contribute observations across multiple months. Patients did not have to be in both pre- and post- periods to be included in the model (*see Sensitivity Analysis*). ITS analysis was used to evaluate changes in SBP associated with the onset of the COVID-19 pandemic, estimating both immediate level changes and differences in pre- and post-onset of pandemic trends [14, 19].

In our primary, unadjusted interrupted time series analyses, we included the following variables: Time (months since January 2017), Pandemic Intervention (binary indicator representing pre- versus post-pandemic onset), and the interaction term (Time * Pandemic Intervention) (see Appendix 1, Equation 1.1). Here, Time represents the pre-pandemic regression slope, while Time * Pandemic Intervention as the difference between post- and pre-pandemic onset slopes. The primary outcome of interest, captured by the Pandemic Intervention coefficient, represents the immediate “level change”—the abrupt shift in SBP coinciding with the pandemic onset in March 2020. This “level change” is used interchangeably with the increase in SBP associated with the pandemic onset. To account for seasonality variation in SBP, we included periodic sine and cosine terms in the regression model. Interrupted time series analyses excluded the transition period between March 2020 to July 2020 due to small sample size from lockdown (See Supplemental Fig. 3). We did, however, include this transition period in our sensitivity analysis discussed below. The intersection of the post-pandemic and pre-pandemic regression lines—derived from the estimated slopes and intercepts of the interrupted time series model—was used to estimate the time point at which modeled systolic blood pressure returned to the level predicted by the pre-pandemic trend. We refer to this point as the “return to pre-pandemic trend,” defined as the month at which the post-pandemic fitted SBP equaled the counterfactual SBP predicted under continuation of the pre-pandemic trend.

In our main adjusted ITS model, we controlled for demographic factors (age, sex, racial/ethnicity group) and pre-existing comorbidities (HTN, obesity, diabetes, CKD, CAD, CHF, HLD, asthma, COPD) (Appendix 1, Equation 1.2). Model assumptions were assessed using visual inspection of residual plots and evaluation of residual autocorrelation.

To examine heterogeneity in pandemic-related SBP changes, we conducted pre-specified stratified ITS analyses based on prior literature and the study’s conceptual framework, including stratification by sex, race/ethnicity, age group, and pre-existing hypertension status. Each model adjusted for all remaining demographic factors and comorbidities (Appendix 1, Equation 1.3a).

For each demographic stratifier, we additionally included the stratifying variable and its interaction with the pandemic intervention in the ITS model to formally test effect modification and to estimate differences in pandemic-related SBP changes relative to a reference group (e.g., female vs male; non-Hispanic Black vs non-Hispanic White; ≥70 vs 18–29 years) (Appendix 1, Equation 1.3b).

We then applied the same analytic framework to neighborhood-level socioeconomic status including ZIP-code–level quintiles of median household income, educational attainment, and unemployment rate. These stratified analyses were adjusted for age, sex, race/ethnicity, and comorbidities, and interaction terms were included to compare each quintile with the highest-SES reference group (Appendix 1, Equations 1.4a and 1.4b).

For all stratifying variables, interaction terms were evaluated and stratified effect estimates with 95% confidence intervals were reported regardless of interaction p-values, because non-significant interaction tests do not preclude clinically meaningful subgroup differences. For all models, a complete-case analysis was used to address missingness in variables included. All statistical analysis was conducted using Python (version 3.9.7) using the *statsmodel* package [20].

### Sensitivity analyses

We assessed robustness by running the fully adjusted model restricted to patients with at least one SBP measurement in both pre- and post- pandemic periods. Additionally, we conducted analyses across four distinct periods: pre-pandemic (before March 2020), early pandemic (March–June 2020), pandemic (August 2020–July 2023), and post-pandemic (after July 2023), to explore differences across these intervals (Equation 1.5).

### Ethics approval and consent to participate

This study was conducted in accordance with the principles outlined in the Declaration of Helsinki and all applicable institutional and regulatory guidelines. Approval was obtained from the Einstein–Montefiore Institutional Review Board (IRB #2021-13658). The requirement for informed consent was waived by the Institutional Review Board due to the retrospective nature of the study and use of de-identified electronic health record data. The Strengthening the Reporting of Observational Studies in Epidemiology (STROBE) reporting guideline was used to guide the reporting of this study.

## Results

Table 1 shows patient demographics for pre- and post-onset of pandemic groups. The study included 558,121 patients pre-onset and 528,815 patients post-onset, with a mean age increasing slightly from 49.7 ± 19.3 years to 51.2 ± 19.4 years, and approximately 60% females in both groups. Racial/ethnic composition remained stable, with Hispanic patients representing the largest group, followed by non-Hispanic Black, non-Hispanic White, and other racial/ethnic groups.

**Table 1:** Patient Demographics, Comorbidities, and social determinants of health between Pre-Pandemic onset (Jan 2017-March 2020) and Post-Pandemic onset (July 2020-Aug 2024).

Variable	Pre-Pandemic Onset (N = 558,121)	Post-Pandemic Onset (N = 528,815)	Standardized Mean Difference
**Age, mean** **±** **SD**	49.72 ± 19.29	51.15 ± 19.44	0.07
**Age group, n (%)**			0.06
18–29	108,440 (19.4)	92,766 (17.5)	
30–39	84,142 (15.1)	76,770 (14.5)	
40–49	78,991 (14.2)	72,652 (13.7)	
50–59	99,567 (17.8)	88,002 (16.6)	
60–69	91,024 (16.3)	95,690 (18.1)	
≥70	95,957 (17.2)	102,935 (19.5)	
**Female sex, n (%)**	345,029 (61.8)	319,974 (60.5)	0.03
**Race/ethnicity, n (%)**			0.04
Non-Hispanic White	76,243 (13.7)	67,982 (12.9)	
Non-Hispanic Black	148,084 (26.5)	137,654 (26.0)	
Hispanic	197,029 (35.3)	196,671 (37.2)	
Other	136,765 (24.5)	126,508 (23.9)	
**Pre-existing conditions, n (%)**			
Asthma	67,787 (12.1)	82,152 (15.5)	0.10
COPD	22,852 (4.1)	27,241 (5.2)	0.05
Diabetes	102,418 (18.4)	116,758 (22.1)	0.09
CHF	29,444 (5.3)	38,810 (7.3)	0.08
HTN	186,417 (33.4)	210,859 (39.9)	0.14
CKD	49,293 (8.8)	57,566 (10.9)	0.07
CAD	51,008 (9.1)	68,114 (12.9)	0.12
Obesity	111,450 (20.0)	140,986 (26.7)	0.16
HLD	134,383 (24.1)	174,237 (32.9)	0.20
**Median income quintile, n (%)**			0.12
1st (<$36,730)	115,933 (20.8)	110,287 (21.2)	
2nd ($36,730–$48,212)	119,932 (21.5)	116,236 (22.3)	
3rd ($48,212–$61,087)	102,997 (18.5)	98,416 (18.9)	
4th ($61,087–$79,129)	107,027 (19.2)	98,874 (19.0)	
5th (≥$79,129)	110,834 (19.9)	97,000 (18.6)	
Missing	1398 (0.3)	8002 (1.5)	
**Education quintile, n (%)**			0.11
1st (<68.8%)	122,915 (22.1)	118,230 (22.7)	
2nd (68.8–75%)	112,362 (20.2)	108,238 (20.8)	
3rd (75–82.1%)	116,950 (21.0)	113,085 (21.7)	
4th (82.1–86.1%)	110,274 (19.8)	102,482 (19.7)	
5th (≥86.1%)	94,741 (17.0)	79,174 (15.2)	
Missing	879 (0.2)	7606 (1.4)	
**Unemployment quintile, n (%)**			0.11
1st (≥12.2%)	111,326 (20.0)	97,172 (18.6)	
2nd (9.9–12.2%)	112,803 (20.2)	104,697 (20.1)	
3rd (8.5–9.9%)	118,257 (21.2)	113,299 (21.7)	
4th (6.5–8.5%)	105,064 (18.9)	101,484 (19.5)	
5th (<6.5%)	109,791 (19.7)	104,556 (20.1)	
Missing	880 (0.2)	7607 (1.4)	

Standardized mean differences (SMDs) were calculated to quantify the magnitude of differences between groups. For continuous variables, SMDs were calculated as the difference in means divided by the pooled standard deviation; for binary variables, as the difference in proportions divided by the pooled standard deviation. For multilevel categorical variables, the maximum absolute SBD across categories is shown. No statistical hypothesis testing was performed for baseline characteristics.

Supplemental Fig. 1 illustrates a flowchart of this study. Prevalence of comorbidities such as hypertension, diabetes, obesity, and cardiovascular diseases notably increased post-onset of the pandemic, and the distribution of socioeconomic factors remained similar across both periods (Supplemental Fig. 4).

**Fig. 1:**
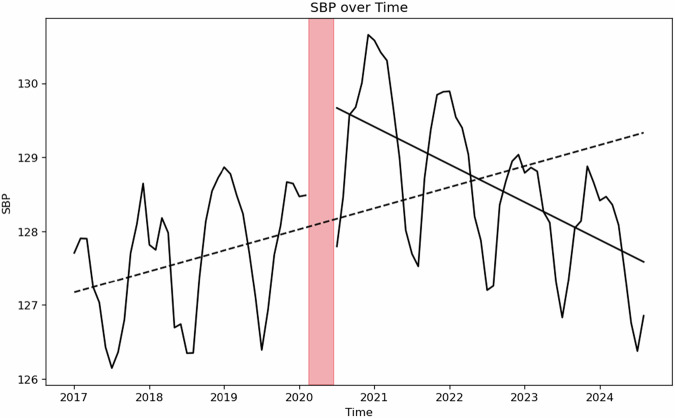
Interrupted time series of outpatient SBP from Jan 2017 to Aug 2024 uncontrolled (See Equation 1.1). Dashed line represents linear regression of pre-pandemic trend. Solid line represents linear regression of post-onset of pandemic trend starting in July 2020. Between March 2020 to July 2020, number of SBP dropped due to shutdown (Supplemental Fig. 3) and data from this period was excluded from fitting. The slope of the dash line is 0.024 mmHg/month and solid line is −0.042 mmHg/month. Intersection of post-onset of pandemic regression line to pre-pandemic line is June 2022. Level changes due to pandemic was 1.76 [1.46,2.06] mmHg.

Figure 1 presents outpatient SBP measurements over time, demonstrating expected seasonal fluctuations, with peaks in winters and troughs in summer, which were adjusted for using sine and cosine functions in subsequent modeling. Unadjusted population-wise, SBP pre-pandemic rose at a rate of 0.02 mmHg (95% C, 0.01-0.03; P < 0.001) per month from 2016 to 2020. Population-wide SBP increased by 1.76 mmHg (95% CI, 1.46–2.06; P < 0.001) associated with the onset of the pandemic and returned to pre-pandemic level by July 2022 (16 months after onset of the COVID-19 pandemic) without controlling for covariates.

Table 2 shows the multivariate analysis controlling for demographics and comorbidities. Population-wide SBP increased by a 1.69 mmHg (95% CI, 1.59–1.78; P < 0.001) associated with pandemic onset. This table further shows stratification by demographic variables and respective level changes, difference in level changes, and p-values after adjusting for other demographic variables and comorbidities. Males and females had non-statistically significant differences in level changes at −0.08 mmHg (95% CI, −0.27–0.12; P = 0.45).

**Table 2:** Level changes in interrupted time series analyses.

	Level change (mmHg)	Difference in level change (mmHg)	p-value
All Patients	1.69 [1.59, 1.78]	–	<0.001
Male	1.78 [1.63,1.93]	–	–
Female	1.68 [1.55,1.8]	−0.08 [−0.27,0.12]	0.45
Non-Hispanic White	1.01 [0.75,1.26]	–	–
Non-Hispanic Black	1.53 [1.34,1.71]	0.55 [0.24,0.86]	<0.001
Hispanic	2.05 [1.89,2.21]	1.10 [0.81,1.40]	<0.001
Other	1.91 [1.69,2.14]	0.92 [0.58,1.26]	<0.001
18–29 y/o	1.73 [1.13,2.33]	–	–
30–39 y/o	1.74 [1.20,2.29]	0.02 [−0.82,0.87]	0.96
40–49 y/o	1.36 [0.91,1.81]	−0.33 [−1.15,0.49]	0.43
50–59 y/o	1.39 [1.05,1.73]	−0.18 [−0.96,0.60]	0.66
60–69 y/o	1.29 [0.98,1.59]	−0.24 [−1.01,0.54]	0.55
70+ y/o	1.56 [1.26,1.85]	0.12 [−0.66,0.91]	0.76
No Pre-Existing HTN	1.68 [1.53,1.83]	–	–
Pre-Existing HTN	1.65 [1.53,1.78]	−0.03 [−0.24,0.18]	0.31

The “All Patients” model is adjusted for demographic factors (age, sex, race/ethnicity) and pre-existing comorbidities. Model is stratified by sex, race/ethnicity, and age groups, with all other variables being controlled for. Each stratified variable is also included as an interaction term to compare to reference group. Time of intersection for each post-onset group to its pre-pandemic trend is also tabulated.

Compared to Non-Hispanic Whites, Non-Hispanic Blacks had 0.55 mmHg (95% CI, 0.24–0.86; P < 0.001) greater increases in SBP, Hispanics had 1.10 mmHg (95% CI, 0.81–1.40; P < 0.001) greater increases in SBP, and Others had 0.92 mmHg (95% CI, 0.58–1.26; P < 0.001) greater increases in SBP. Compared to the 18–29 y/o age group, other age groups had non-statistically significant differences in level changes. There was no statistically significant difference in SBP increases across pre-existing HTN status.

Table 3 shows the level SBP changes stratified by zip-code level median income, education, and unemployment--controlling for age, sex, race/ethnicity, and comorbidities. Patients from lower median income zip codes exhibited greater level changes in SBP associated with pandemic onset as compared to the highest quintile. Those living in a zip code with a median income less than $36730 (1^st^ quintile) experienced a 1.77 mmHg (95% CI, 1.60–1.94) increase in SBP while those living in a median income greater than $79129 (5^th^ quintile) experienced a 1.23 mmHg (95% CI, 1.03–1.43) increase. When incorporating the interaction term, the 1^st^ quintile had 0.58 mmHg (95% CI, 0.32–0.85; P < 0.001) greater increase in SBP compared to the 5^th^ quintile. All other quintiles experienced greater SBP increases when compared to the 5^th^ quintile. Similar trends were seen with education and unemployment trends.

**Table 3:** Level changes and associated p-values of stratified groups based on median income quintile, education quintile, and median unemployment quintiles derived from zip-code level data.

	Level Change (mmHg)	Difference in Level Change (mmHg)	p-value
**Median Income Quintiles**			
1st quintile (<$36730)	1.77 [1.6,1.94]	0.58 [0.32,0.85]	<0.001
2nd quintile ($36730-$48212)	1.98 [1.81,2.14]	0.81 [0.55,1.07]	<0.001
3rd quintile ($48212-$61087)	1.93 [1.76,2.11]	0.79 [0.52,1.05]	<0.001
4th quintile ($61087-$79129)	1.51 [1.33,1.69]	0.31 [0.04,0.58]	0.02
5th quintile ($79129+)	1.23 [1.03,1.43]	–	–
**Education Quintiles**			
1st quintile (<68.8%)	1.85 [1.68,2.01]	0.72 [0.45,1]	<0.001
2nd quintile (68.8–75%)	1.93 [1.76,2.1]	0.81 [0.54,1.09]	<0.001
3rd quintile (75–82.1%)	1.68 [1.51,1.86]	0.57 [0.29,0.85]	<0.001
4th quintile (82.1–86.1%)	1.67 [1.49,1.84]	0.57 [0.29,0.85]	<0.001
5th quintile (86.1%+)	1.22 [1.01,1.44]	–	–
**Median Unemployment Quintiles**			
1st quintile (12.2%+)	1.86 [1.69,2.04]	0.67 [0.4,0.93]	<0.001
2nd quintile (9.9–12.2%)	1.8 [1.62,1.98]	0.62 [0.35,0.88]	<0.001
3rd quintile (8.5–9.9%)	1.94 [1.77,2.1]	0.76 [0.5,1.02]	<0.001
4th quintile (6.5–8.5%)	1.63 [1.45,1.8]	0.44 [0.18,0.7]	0.001
5th quintile (<6.5%)	1.24 [1.04,1.44]	–	–

Lower quintiles is associated with lower SES. Model is controlled for age, sex, race/ethnicity, and comorbidities. Each stratified variable is also included as an interaction term to compare to reference group.

### Sensitivity analysis

Sensitivity analyses restricted to individuals with SBP measurements in both pre- and post-pandemic periods (n = 290,166) yielded similar SBP increases at 1.59 mmHg (95% CI, 1.49–1.69). Further analyses dividing the study into distinct periods showed a slight decrease during the early pandemic period at −0.25 mmHg (95% CI, −0.47–−0.03), but significantly higher increases during the pandemic at 2.14 mmHg (95% CI, 2.04–2.25) and post-pandemic periods at 2.53 mmHg (95% CI, 1.93–3.13) relative to pre-pandemic trends.

## Discussion

This study examined outpatient systolic blood pressure in an urban population in the Bronx across the COVID-19 pandemic from Jan 2017 to Aug 2024. The major findings were: 1) outpatient SBP rose by 1.76 mmHg (95% CI, 1.46–2.06) and 1.69 mmHg (95% CI, 1.59–1.78), without and with adjustment for confounders, respectively, at the onset of pandemic, 2) outpatient SBP did not return to pre-pandemic level until 16 months later (July 2022), 3) non-Hispanic Black, Hispanic, and other racial/ethnic groups experiences greater SBP increases compared with non-Hispanic Whites; and 4) individuals from lower socioeconomic status zip codes (lower median incomes, education levels, and higher unemployment rates) showed significantly larger SBP increases compared to their higher SES counterparts.

### Prior studies

The rise in population-level outpatient SBP in early pandemic is consistent with a few prior studies. Laffin et al. documented SBP increases of 1.10–2.50 mmHg among U.S. adults in 2020 compared with 2019.^12^ Nolde et al. reported similar upward shifts in blood pressure within the Australian healthcare system and Gotanda et al. observed increases among hypertensive patients during the initial months of the pandemic [11, 12, 14].

Extending these findings, our analysis demonstrates that pandemic-associated SBP increases persisted beyond the early pandemic period and gradually returned toward pre-pandemic trends over time, with substantial heterogeneity across racial/ethnic and socioeconomic groups. These results suggest that population-level blood pressure changes during large-scale disruptions may have prolonged cardiometabolic implications, particularly for populations already experiencing structural disadvantages.

### Potential causes

The pandemic-associated increase in SBP likely reflects a combination of healthcare disruptions and broader stressors, with disproportionate impacts on racial and ethnic minority populations and individuals with lower socioeconomic status, groups that already experience higher hypertension burden and barriers to care. Prior studies have shown that these populations faced greater disruptions in outpatient care, reduced continuity of chronic disease management, and heightened financial and psychosocial stress during the pandemic, providing important context for the observed heterogeneity in pandemic-associated SBP changes across demographic and socioeconomic groups. These structural inequities have been linked to worsening cardiovascular risk profiles, including blood pressure control, and may help contextualize the amplified SBP changes observed in our study among non-Hispanic Black and Hispanic populations. Our findings extend this literature by demonstrating that these disparities were evident not only in early pandemic periods but persisted over time at the population level [2, 3, 6, 21–26].

Consistent with this context, our stratified analyses showed larger pandemic-associated SBP increases among racial/ethnic minority groups and residents of lower-income, lower-education, and higher-unemployment ZIP codes, while no significant differences were observed by age, sex, or pre-existing hypertension status.

### Public health implications

Although population SBP eventually returned to pre-pandemic trends, it took 16 months to return to pre-pandemic level and the elevated SBP might have already exerted negative health impact on at-risk individuals. Our findings provide important insights into the temporal dynamics of population-wide SBP across the COVID-19 pandemic.

Modest increases in population-wide blood pressure can have serious long-term public health implications due to the strong relationship between elevated blood pressure and cardiovascular diseases. Prior studies have shown that a 2 mmHg rise in SBP at the population level is associated with a 7% increase in ischemic heart disease mortality and a 10% rise in stroke mortality [27]. These small changes at the population can also lead to a substantial increase in future cardiovascular diseases and related complications. Even more, short-term and medium-term variability in BP has recently been shown to lead to higher all-cause mortality and cardiovascular disease risk [28, 29].

A higher SBP exerts stress on the arteries, leading to endothelial dysfunction, vascular remodeling, and atherosclerosis, which elevate the risks of coronary artery disease, heart failure, and stroke [30]. Small-vessel damage in the brain, caused by elevated SBP, is linked to a higher likelihood of stroke and cognitive decline [31].

Given the sheer number of individuals infected by SARS-CoV-2 worldwide, our findings have important public health implications, emphasizing the need for more diligent blood screening of at-risk population and targeted hypertension management strategies in the post-pandemic era. Efforts to improve blood pressure control should focus on expanding access to routine monitoring and care, especially for underserved populations, and addressing the social determinants of health that underlie disparities in blood pressure outcomes.

Moreover, the disparities in increases in SBP levels among certain groups suggest that the pandemic may have induced lasting changes in cardiovascular risk profiles, necessitating ongoing surveillance and support. Public health policies should prioritize enhancing resilience in the healthcare system to prevent similar disruptions in chronic disease management and introducing environmental stressors during future crises. Finally, we noted that negative impact of COVID-19 pandemic on increased incidence of new asthma and asthma exacerbation [32], trends of HbA1c [33], as well as upstaging of breast cancer diagnosis [34] have been reported.

### Limitations

This study has several limitations. Our findings were limited to only patients who returned to our health system for any reason, including routine checkups. Although the Montefiore Health System is the predominant health system with multiple hospitals and outpatient clinics in the Bronx, these data came from a single health system that serves a predominantly urban, low-income, and racially and ethnically diverse population in the Bronx. As a result, the magnitude and timing of pandemic-related disruptions—and their downstream effects on blood pressure—may differ from those observed in regions that experienced later or less intense surges. Populations with higher baseline socioeconomic resources, more stable access to healthcare, or greater capacity for remote work may have experienced smaller or more transient changes in blood pressure. In addition, urban environments may differ from suburban or rural settings with respect to population density, healthcare access, stress exposures, and pandemic mitigation measures, which could influence blood pressure trajectories. While these factors may limit direct generalizability, the study’s focus on an early epicenter provides important insight into how large-scale public health disruptions can affect cardiometabolic risk in vulnerable populations and may represent an upper bound of pandemic-related impact.

We performed a few sensitivity analyses. Notably, we tested the robustness of our findings by restricting the analysis to individuals with SBP measurements both before and after the pandemic onset. Sensitivity analyses by segmenting different pandemic periods revealed a modest decline during the early pandemic, likely related to clinic closures, followed by significant increases during the main pandemic period and post-pandemic period. These trends suggest prolonged disruptions in healthcare access and lifestyle factors contributed to persistent SBP elevation. Although we have adjusted for major confounds, there is potential for both sampling and method-based biases. For example, individual SARS-CoV-2 infection status and antihypertensive medication adherence or prescription fill patterns were not modeled. Therefore, observed population-level SBP changes may reflect a combination of pandemic-related stressors, healthcare disruptions, lifestyle changes, reduced medication adherence, possible direct cardiovascular effects of infection, and unmeasured changes in clinical practice or measurement methods during the pandemic. While digital healthcare utilization was not accounted for in our analyses, we acknowledge that access and use of digitally enabled healthcare services before, during, and after the pandemic may have significantly impacted BP outcomes.

Additionally, our primary interrupted time series analyses treated patient-month observations as independent, despite repeated measurements contributed by the same individuals over time. Although monthly averaging reduces short-term within-person variability, residual within-person correlation may persist and could lead to underestimation of standard errors and overconfidence in p-values. In addition, residual serial autocorrelation may remain even after adjustment for seasonality using sine and cosine terms. Because standard regression models assume independent errors, any remaining within-person or temporal autocorrelation could result in overly optimistic statistical inference; accordingly, results should be interpreted with appropriate caution.

Socioeconomic status was assessed using ZIP-code–level indicators derived from census data, which reflect neighborhood context rather than individual-level socioeconomic circumstances. Substantial heterogeneity may exist within ZIP codes, and assigning area-level averages to individuals may result in misclassification and ecological fallacy.

## Conclusions and future perspectives

Our findings provide valuable insights into the acute and evolving impacts of the COVID-19 pandemic on population health with respect to systolic blood pressure. Population-level outpatient SBP rose abruptly during the early pandemic and gradually returned to pre-pandemic levels after 16 months. Additionally, SBP increases were more pronounced in those who lived in lower SES zip codes and racial/ethnic minorities. The rise in SBP likely reflected the combined effects of stressors, including psychological stress, fear, social isolation, disrupted healthcare access, and possibly direct impacts of SARS-CoV-2 infection. The rise in SBP at the population level likely has long-term health consequences that warrant further investigation. The gradual return to baseline highlights the healthcare system’s resilience and the adaptive capacity of individuals as conditions stabilized. Beyond documenting a transient rise in SBP, the study quantifies the magnitude and trajectory of these changes among various sociodemographic groups, offering a framework for understanding cardiovascular risks during future public health crises. These findings underscore the complex interplay between healthcare disruptions, social determinants of health, and hypertension control, emphasizing the importance of maintaining continuity of care, addressing stress-related health impacts, and implementing targeted interventions to mitigate pandemic-related health disparities and long-term health consequences.

## Summary Table

### *What is known about the topic:*

- Average population SBP and percentage of uncontrolled SBP among hypertensive patients rose during the pandemic.
- Neighborhood-level socioeconomic status is linked with worse cardiovascular outcomes.

### *What this study adds:*

- This study examines the impact of the pandemic on outpatient SBP and examines the time period of when these trends return to baseline.
- Racial/ethnic minorities and lower socioeconomic status were associated with larger increases in SBP during the pandemic, which may lead to negative long-term cardiovascular outcomes and exacerbating pre-existing disparities.

## Supplementary information

Appendix

Supplemental Figure 1

Supplemental Figure 2

Supplemental Figure 3

Supplemental Figure 4

## Data Sharing Statement

Data used is available from the corresponding author upon reasonable request.
