# Supplementary material for: Blood pressure trends and disparities across the COVID-19 pandemic in a large diverse urban population
Source: J Hum Hypertens. 2026 Mar 13;40(4):311–8. doi: 10.1038/s41371-026-01130-z (PMC13068518; doi:10.1038/s41371-026-01130-z)
Supplement: Supplementary file 1 — Appendix [file 41371_2026_1130_MOESM1_ESM.docx]

**Appendix 1.**

Equation 1.1 (Uncontrolled Main Analysis):

SBP=β0​ + β1 ​× Time + β2 ​× Pandemic Intervention + β3 ​× (Time * Pandemic Intervention) + β4 × sin (2π × Time / 12) + β5 × cos (2π × Time / 12)

Equation 1.2 (Controlled Main Analysis):

SBP=β0​ + β1 ​× Time + β2 ​× Pandemic Intervention + β3 ​× (Time * Pandemic Intervention) + β4 × sin (2π × Time / 12) + β5 × cos (2π × Time / 12) + β6 ​× Age + β7 ​× Sex + β8 ​× Non_Hispanic_Black + β9 ​× Hispanic + β10 ​× Other + β11-20 ​× Comorbidity List

Equation 1.3a (Stratified by Given Demographic [e.g. sex]):

For sex=0 and sex=1:

SBP=β0​ + β1 ​× Time + β2 ​× Pandemic Intervention + β3 ​× (Time * Pandemic Intervention) + β4 × sin (2π × Time / 12) + β5 × cos (2π × Time / 12) + β6 ​× Age + β7 ​× Non_Hispanic_Black + β8 ​× Hispanic + β9 ​× Other + β10-19 ​× Comorbidity List

Equation 1.3b (Including Given Demographic with Interaction Term [e.g. sex])

SBP=β0​ + β1 ​× Time + β2 ​× Pandemic Intervention + β3 ​× (Time * Pandemic Intervention) + β4 × sin (2π × Time / 12) + β5 × cos (2π × Time / 12) + β6 ​× Age + β7 ​× Sex + β8 ​× Non_Hispanic_Black + β9 ​× Hispanic + β10 ​× Other + β11-20 ​× Comorbidity List + β21 ​× (Sex * Time) + β22 ​× (Sex * Pandemic Intervention) + β23 ​× (Sex * Time * Pandemic Intervention)

Equation 1.4a (Stratified by Quintile [e.g. median income=3]):

For quintile=3:

SBP=β0​ + β1 ​× Time + β2 ​× Pandemic Intervention + β3 ​× (Time * Pandemic Intervention) + β4 × sin (2π × Time / 12) + β5 × cos (2π × Time / 12) + β6 ​× Age + β7 ​× Sex + β8 ​× Non_Hispanic_Black + β9 ​× Hispanic + β10 ​× Other + β11-20 ​× Comorbidity List

Equation 1.4b (Including Quintile with Interaction Term Compared to Reference Quintile [e.g. median income=3]):

For quintile =5 (Reference) OR quintile = 3

SBP=β0​ + β1 ​× Time + β2 ​× Pandemic Intervention + β3 ​× (Time * Pandemic Intervention) + β4 × sin (2π × Time / 12) + β5 × cos (2π × Time / 12) + β6 ​× Age + β7 ​× Sex + β8 ​× Non_Hispanic_Black + β9 ​× Hispanic + β10 ​× Other + β11-20 ​× Comorbidity List + β21 ​× isQuintile3 + β22 (isQuintile3 * Time) + β23 ​× (isQuintile3Pandemic Intervention) + β23 ​× (isQuintile3 * Time * Pandemic Intervention)

Equation 1.5 (Dividing Time into Pre-, Early-, During-, Post- Pandemic)

SBP=β0​ + β1 ​× Time + β2 ​× Pandemic Intervention + β3 × sin (2π × Time / 12) + β4 × cos (2π × Time / 12) + β5 ​× Age + β6 × Sex + β7 ​× Non_Hispanic_Black + β8 ​× Hispanic + β9 ​× Other + β10-19 ​× Comorbidity List + β20 ​× Early Pandemic + β21 ​× During Pandemic + β22 ​× After Pandemic + β23 × (Early Pandemic * Time) + β24 × (During Pandemic * Time) + β25 × (After Pandemic * Time)
