# Supplementary material for: Blood pressure trends and disparities across the COVID-19 pandemic in a large diverse urban population
Source: J Hum Hypertens. 2026 Mar 13;40(4):311–8. doi: 10.1038/s41371-026-01130-z (PMC13068518; doi:10.1038/s41371-026-01130-z)
Supplement: Supplementary file 2 — Supplemental Figure 1 [file 41371_2026_1130_MOESM2_ESM.docx]

**Supplemental Figure 1: Study flowchart. An eligible population was included in the study cohort based on pre-pandemic onset and post-pandemic onset time periods of SBP measurements.**
