# Supplementary material for: Blood pressure trends and disparities across the COVID-19 pandemic in a large diverse urban population
Source: J Hum Hypertens. 2026 Mar 13;40(4):311–8. doi: 10.1038/s41371-026-01130-z (PMC13068518; doi:10.1038/s41371-026-01130-z)
Supplement: Supplementary file 3 — Supplemental Figure 2 [file 41371_2026_1130_MOESM3_ESM.docx]

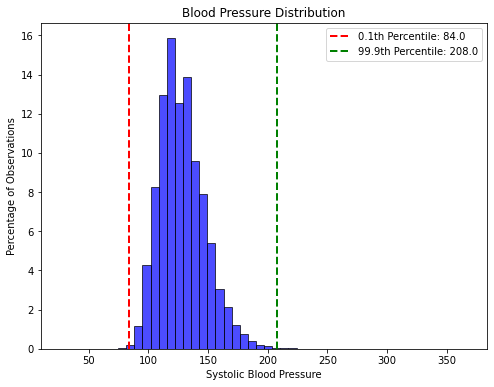


***Supplemental Figure 2:*** *Blood pressure distribution across entire Montefiore Health System data from January 2017 to August 2024. 0.1% and 99.9% thresholds [84.0, 208.0] mmHg were established as SBP cutoffs.*
