# Supplementary material for: Blood pressure trends and disparities across the COVID-19 pandemic in a large diverse urban population
Source: J Hum Hypertens. 2026 Mar 13;40(4):311–8. doi: 10.1038/s41371-026-01130-z (PMC13068518; doi:10.1038/s41371-026-01130-z)
Supplement: Supplementary file 4 — Supplemental Figure 3 [file 41371_2026_1130_MOESM4_ESM.docx]

A)


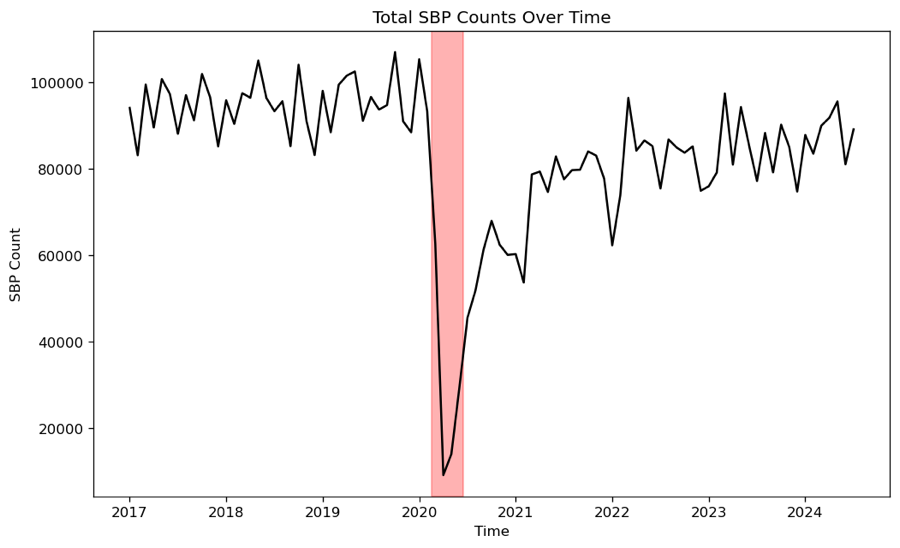


B)


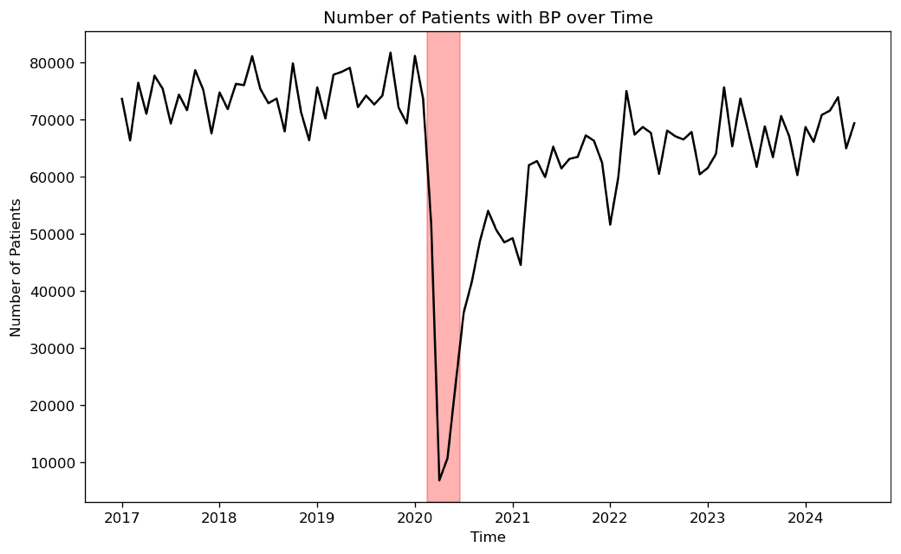


C)


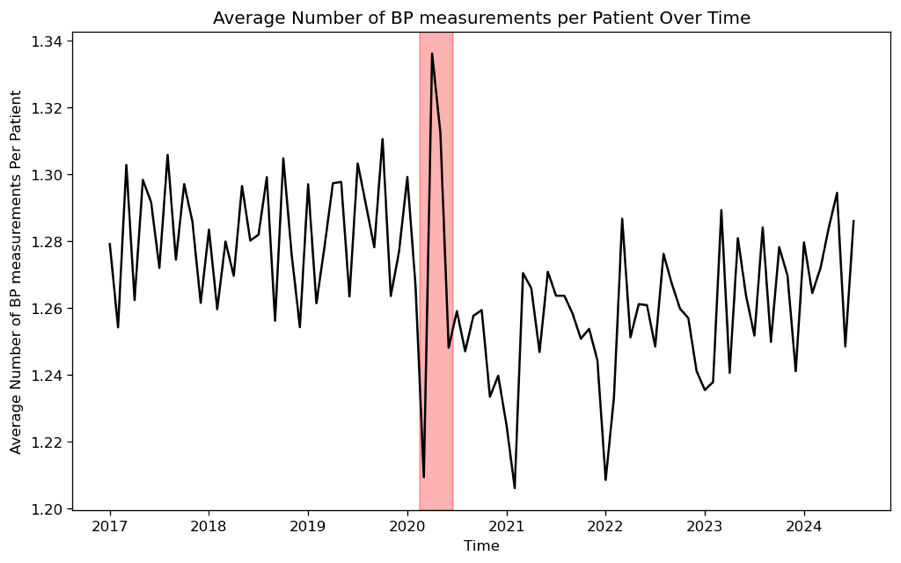


***Supplemental Figure 3:*** *Counts of Total SBP per month over time (A), Patients with SBP Measurements per month over time (B), and Average BP per patient per month over time (C). Outpatient SBP measurement counts dropped precipitously at the onset of pandemic due to lockdowns. Shaded red box represents area excluded in analysis.*
