# Supplementary material for: Blood pressure trends and disparities across the COVID-19 pandemic in a large diverse urban population
Source: J Hum Hypertens. 2026 Mar 13;40(4):311–8. doi: 10.1038/s41371-026-01130-z (PMC13068518; doi:10.1038/s41371-026-01130-z)
Supplement: Supplementary file 5 — Supplemental Figure 4 [file 41371_2026_1130_MOESM5_ESM.docx]

*A)*


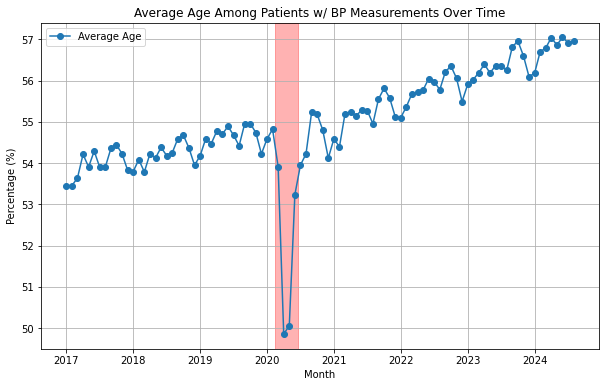


*B)*


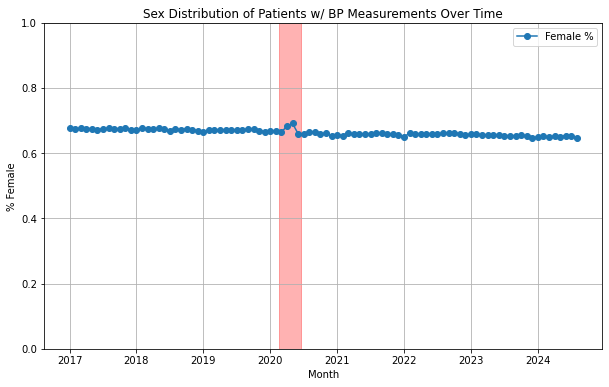


*C)*


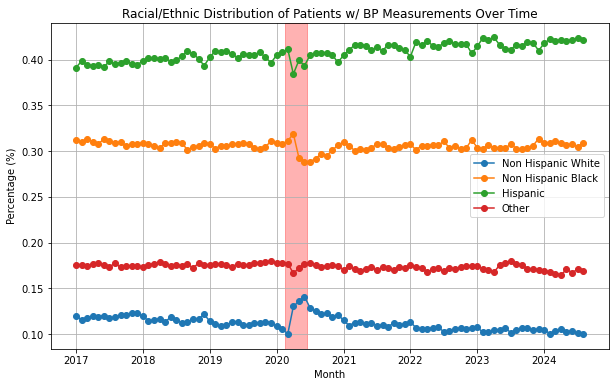


*D)*

*
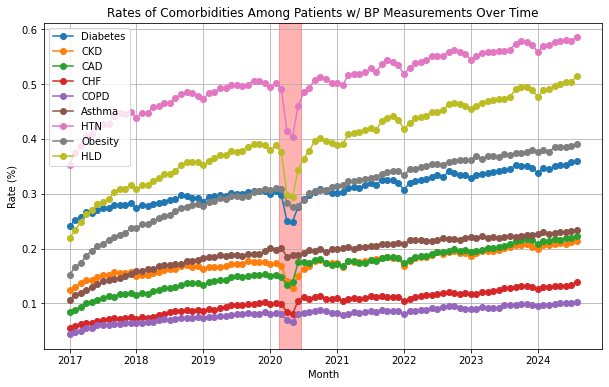
*

***Supplemental Figure 4****: Patient characteristics including age (A), sex (B), racial/ethnic group (C), and pre-existing comorbidities (D) across duration of study (January 2017-August 2024) among those with valid SBP measurements.*
